# Real-World Evidence on Different Amoxicillin-Containing Regimens for *Helicobacter pylori* Treatment in Elderly Patients: Analysis of Efficacy, Safety, and Virulence Gene Association

**DOI:** 10.3390/antibiotics15040355

**Published:** 2026-03-30

**Authors:** Xue-Liang Chen, Wen Gao, Hui Ye, Zi-Cheng Wang, Hong Cheng, Xue-Zhi Zhang

**Affiliations:** 1 Department of Traditional Chinese Medicine and Integrative Medicine, Peking University First Hospital, Beijing 100034, China; 19186958574@163.com (X.-L.C.); brigh-leaf723@163.com (H.Y.); wzc13909782623@icloud.com (Z.-C.W.); 2 Department of Gastroenterology, Peking University First Hospital, Beijing 100034, China; gaowen@pku.edu.cn

**Keywords:** amoxicillin, vonoprazan, *Helicobacter pylori*, elderly, virulence genes

## Abstract

**Background:** *Helicobacter pylori* (*H. pylori*) infection is an established risk factor for gastric cancer. However, treatment efficacy and the underlying mechanisms in elderly patients with *H. pylori* infection remain incompletely characterised. This study aimed to compare the eradication efficacy and safety of four amoxicillin-containing regimens for *H. pylori* infection in elderly patients. **Methods**: Elderly patients (age ≥ 60 years) with *Helicobacter pylori* infection treated at five hospitals in Beijing between January 2018 and June 2025 were enrolled. Participants were stratified into four groups according to the prescribed regimen: vonoprazan–amoxicillin (VA) dual therapy, rabeprazole–amoxicillin (RA) dual therapy, rabeprazole–Jinghua Weikang (a Chinese herbal medicine, granules)–amoxicillin–furazolidone (RJAF) quadruple therapy, and rabeprazole–bismuth–amoxicillin–furazolidone (RBAF) quadruple therapy. The primary endpoint was the eradication rate for each regimen. Secondary outcomes included the incidence of adverse events (AEs) and data on comorbidities. In addition, serological testing for *H. pylori* virulence-associated antibodies (CagA, VacA, UreA, and UreB) was performed in 32 patients at baseline, prior to treatment initiation. **Results**: A total of 312 patients were screened. The eradication rates with VA, RA, RJAF, and RBAF were 96.3%, 94.0%, 86.8%, and 86.6%, respectively (χ^2^ = 6.92, *p* = 0.075). The incidence of AEs was 13.8%, 15.5%, 17.9%, and 19.1% in the VA, RA, RJAF, and RBAF groups, respectively (*p* = 0.391). **Conclusions**: In elderly patients with *Helicobacter pylori* infection, dual therapy demonstrates non-inferior efficacy compared with triple therapy and conventional quadruple therapy. More complex regimens do not confer additional clinical benefit. Among the two dual-therapy regimens, VA dual therapy shows superior overall performance and is therefore recommended as the first-line treatment of choice.

## 1. Introduction

*Helicobacter pylori* (*H. pylori*) infection is among the most prevalent chronic infectious diseases worldwide and represents a major pathogenic factor in a spectrum of gastrointestinal disorders, including chronic gastritis, peptic ulcer disease, and gastric cancer. In developing countries, the prevalence exceeds 50% [1].

In elderly patients with *H. pylori* infection, eradication therapy may delay the progression of premalignant gastric lesions, such as mucosal atrophy and intestinal metaplasia, thereby reducing gastric cancer risk [2]. However, owing to age-related physiological decline, a higher burden of comorbidities, and reduced drug tolerance, older adults are more vulnerable to treatment-related adverse events (AEs), including gastrointestinal disturbances, increased hepatic and renal burden, and gut microbiota dysbiosis [3,4]. In addition, the increasing prevalence of antibiotic-resistant *H. pylori* strains and suboptimal medication adherence in elderly patients may contribute to eradication failure.

Amoxicillin is a commonly used antibiotic in *H. pylori* eradication therapy. Globally, *H. pylori* resistance to amoxicillin is substantially lower than resistance to other frequently used agents, such as clarithromycin and metronidazole [5]. Furthermore, amoxicillin has a broad antibacterial spectrum and a favourable safety profile [6]. Consequently, it is often combined with proton pump inhibitors (PPIs), bismuth-containing agents, or traditional Chinese medicine (TCM) preparations to constitute various eradication regimens. PPIs exert their principal pharmacological effect by inhibiting the gastric parietal cell H^+^/K^+^-ATPase, thereby significantly reducing intragastric acidity. This creates an intragastric milieu that enhances the antibacterial activity of antibiotics such as amoxicillin, partially suppresses bacterial proliferation, and concurrently alleviates acid-related dyspeptic symptoms associated with *H. pylori* infection [7]. Jinghua Weikang, a commonly used TCM preparation for anti-*H. pylori* therapy, has been reported to improve overall eradication effectiveness and symptom control when combined with amoxicillin-based dual or triple therapy. It also reduces recurrence, thereby enhancing patients’ quality of life. Additionally, it may represent an alternative option for patients with contraindications to bismuth or intolerance [8].

Although studies on the treatment of *H. pylori* infections are abundant both domestically and internationally, there remains a substantial evidence gap in elderly individuals (>60 years). Most available studies focus on the general adult population and do not adequately consider age-related physiological characteristics, resulting in limited evidence to guide optimal treatment strategies for elderly patients in routine practice. Accordingly, this study systematically compared the efficacy and safety of four amoxicillin-containing regimens and proposed the hypothesis that, in eradication therapy for elderly infected patients, amoxicillin-based dual therapy is non-inferior to triple therapy and conventional quadruple therapy, and that more complex regimens do not provide additional therapeutic value.

## 2. Results

### 2.1. Baseline Characteristics Comparison of the Four Patient Groups

**Figure 1:**
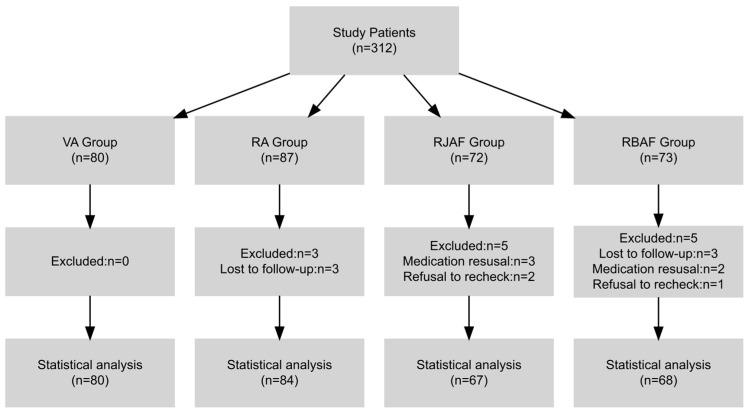
Flowchart of patient screening and recruitment.

A total of 312 *H. pylori*-infected patients aged ≥60 years were assessed, including 80 in the VA group, 87 in the RA group, 72 in the RJAF group, and 73 in the RBAF group. Thirteen patients were excluded due to loss to follow-up or failure to complete the treatment course. The final analysis included 299 patients: 80 in the VA group, 84 in the RA group, 67 in the RJAF group, and 68 in the RBAF group (Figure 1). No significant differences were observed in age, sex, underlying diseases, or baseline urea breath test (UBT) values (all *p* > 0.05). Baseline characteristics were well balanced and comparable across the four groups (Table 1).

### 2.2. Comparison of H. pylori Eradication Efficacy Among the Four Groups

#### 2.2.1. Eradication Rates

**Figure 2:**
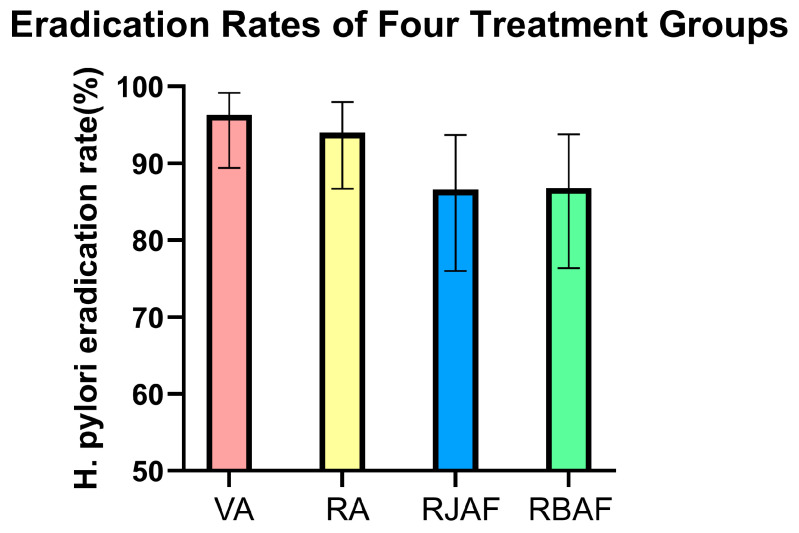
Eradication rates of the four treatment groups.

The *H. pylori* eradication outcomes for the four groups are shown in Figure 2. The eradication rate was 96.3% (77/80, 95% CI: 89.4–99.2%) in the VA group and 94% (79/84, 95% CI: 86.7–98%) in the RA group, with 3 patients lost to follow-up in the RA group. In the RJAF group, the eradication rate was 86.6% (58/67, 95% CI: 76–93.7%); 3 patients discontinued treatment due to adverse reactions, and 2 patients declined follow-up testing. In the RBAF group, the eradication rate was 86.8% (59/68, 95% CI: 76.4–93.8%); 2 patients discontinued due to adverse reactions, 2 were lost to follow-up, and 1 declined follow-up testing. Chi-square test results indicated that the difference in eradication rates among the four groups was not statistically significant (χ^2^ = 6.92, *p* = 0.075).

#### 2.2.2. Changes in UBT Values

The distribution of ΔUBT values before and after treatment across the four groups did not conform to a normal distribution (Kolmogorov–Smirnov test, *p* < 0.05). Therefore, non-parametric tests were employed for analysis. The results showed that the median and interquartile ranges (IQRs) of ΔUBT values were as follows: VA group [18.50 (11.43–24.58)‰], RA group [19.30 (11.83–37.98)‰], RJAF group [19.00 (10.90–39.30)‰], and RBAF group [21.64 (8.63–35.98)‰]. The Kruskal–Wallis H test indicated no statistically significant difference in ΔUBT values among the four groups (H = 1.158, *p* = 0.763).

### 2.3. Incidence of AEs in the Four Patient Groups

Among the total of 299 patients, AEs were reported in 49 cases (16.4%). The incidence of AEs was 13.8% (11/80; 95% CI: 7.1–23.3%) in the VA group, 15.5% (13/84; 95% CI: 8.5–25%) in the RA group, 17.9% (12/67; 95% CI: 9.6–29.2%) in the RJAF group, and 19.1% (13/68; 95% CI: 10.6–30.5%) in the RBAF group.

**Figure 3:**
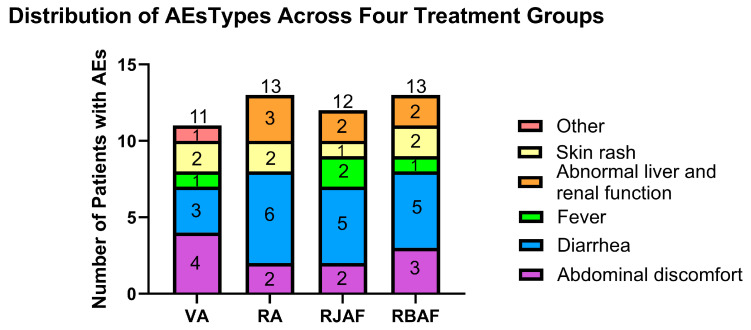
Distribution of AE types across the four treatment groups.

All reported AEs were predominantly mild gastrointestinal symptoms (abdominal discomfort, diarrhoea), with a small number of cases also involving abnormal liver or renal function, rash, or fever (Figure 3). No severe AEs occurred. A total of five patients discontinued treatment due to AEs: three from the RJAF group and two from the RBAF group.

### 2.4. Multivariable Logistic Regression for Predictors of H. pylori Eradication Success

A multivariable logistic regression model was fitted with *H. pylori* eradication status as the dependent variable (failure = 0; success = 1). Covariates included age, sex, underlying diseases, treatment regimen, and the baseline urea breath test (UBT) bacterial-load category. Statistical significance was defined as α = 0.05. The Hosmer–Lemeshow test indicated good model fit (χ^2^ = 10.054, df = 8, *p* = 0.261), supporting adequate model calibration.

**Figure 4:**
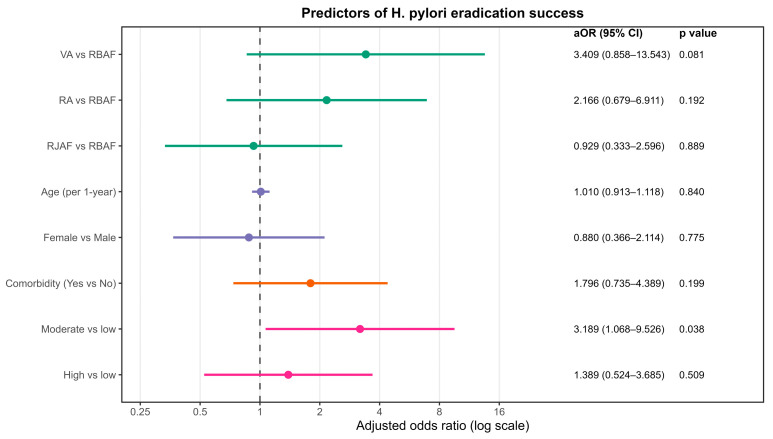
Predictors of *H. pylori* eradication success. Adjusted odds ratios (aOR) and 95% confidence intervals (CI) are presented on a logarithmic scale. horizontal bars represent 95% CIs; the dashed vertical line indicates the null effect (aOR = 1); colored dots denote aOR point estimates for each predictor.

In the adjusted analysis, only a moderate baseline bacterial load was independently associated with eradication success. Compared with the low-bacterial-load category, the moderate-load group had higher odds of successful eradication (OR = 3.189, 95% CI 1.068–9.526; *p* = 0.038). There was no significant difference between the high-load group and the low-load group (OR = 1.389, 95% CI 0.524–3.685; *p* = 0.509). None of the other covariates (age, sex, underlying diseases, or treatment regimen) was independently associated with eradication success (*p* > 0.05). Full regression outputs are provided in Figure 4.

### 2.5. Antibody Testing and Virulence Genes

Serological antibody data were available for 32 patients. Of these, 25 (78.13%) were classified as *H. pylori* type I (CagA and/or VacA positive) and 7 (21.87%) as type II (both CagA and VacA negative). Eradication rates were as follows: CagA-positive (*n* = 22), 95.5% (21/22) versus CagA-negative (*n* = 10), 70.0% (7/10); VacA-positive (*n* = 7), 100.0% (7/7) versus VacA-negative (*n* = 25), 84.0% (21/25); UreA-positive (*n* = 18), 77.8% (14/18) versus UreA-negative (*n* = 14), 100.0% (14/14); UreB-positive (*n* = 12), 83.3% (10/12) versus UreB-negative (*n* = 20), 90.0% (18/20). The eradication rate was 100.0% (3/3) in the VacA + CagA double-positive group and 66.7% (4/6) in the UreA + UreB double-positive group.

Given the small sample size, Fisher’s exact test was used. No statistically significant differences in eradication rates were observed between antibody-positive and antibody-negative groups for any individual antibody (all *p* > 0.05). In addition, the difference in eradication rates between the two composite antibody groups was not statistically significant (Fisher’s exact test, *p* = 0.500).

## 3. Discussion

Using real-world data, our study systematically compared the effectiveness and safety of four amoxicillin-containing regimens for *H. pylori* eradication in older adults. Given its low resistance rates and favourable safety profile, amoxicillin is widely used as a first-line component of *H. pylori* eradication therapy [5], and multiple studies have evaluated regimens in which amoxicillin serves as the backbone antibiotic [9]. In the present study, amoxicillin-based dual therapy (VA and RA groups) achieved eradication rates > 90%, exceeding those observed with rabeprazole plus Jinghua Weikang quadruple therapy and with bismuth-containing quadruple therapy. This is consistent with most previous reports [10,11].

Multiple meta-analyses and network meta-analyses from China and elsewhere have provided evidence supporting the advantages of dual therapy [12,13]. High-dose dual therapy has shown non-inferior eradication rates compared with conventional triple or quadruple regimens while offering advantages in the consistency of treatment efficacy and in adverse-event (AE) control. Dual therapy has also demonstrated effectiveness and safety as rescue treatment for *H. pylori*, particularly in refractory infections or in clarithromycin-resistant strains [14].

Additionally, several exploratory studies have focused on optimising amoxicillin dosing [15,16]. Evidence suggests that vonoprazan plus amoxicillin 1 g twice daily (bid) is non-inferior for *H. pylori* eradication compared with amoxicillin 1 g three times daily (tid) [17]. This has important clinical implications for older adults, especially those of advanced age or with low body weight, who often have reduced physiological reserve and poorer drug tolerance. Appropriately reducing the amoxicillin dose, while maintaining eradication efficacy, may further decrease the risk of AEs (notably gastrointestinal adverse effects) and thereby improve adherence.

During *H. pylori* eradication in older adults, drug safety warrants attention alongside efficacy. In our study, all four amoxicillin-containing regimens demonstrated a favourable safety profile. Reported AEs were predominantly mild gastrointestinal symptoms, and no severe hepatic or renal dysfunction was observed. Diarrhoea was the most common AE across all four regimens, typically presenting as mild-to-moderate loose stools, with only a minority reporting watery stools. This was followed by abdominal discomfort, mainly bloating and pain. In addition, one patient experienced blood pressure fluctuations that improved after discontinuation of antihypertensive therapy; this was considered related to underlying essential hypertension.

The mechanisms underlying these AEs, beyond direct gastrointestinal mucosal irritation and potential drug–drug interactions, may importantly involve treatment-related perturbations of the gut microbiota. As a broad-spectrum β-lactam antibiotic, amoxicillin targets *H. pylori* but also non-selectively suppresses commensal intestinal bacteria, thereby disrupting microbial homeostasis—an effect that may be particularly evident in the short term. A 2022 clinical study reported that amoxicillin-containing regimens alter gut microbial diversity and community structure [18]. Recovery to baseline may take at least 9 months, and such perturbations have been associated with a marked increase in the diversity of antibiotic-resistance genes, as well as indirect effects on the intestinal mycobiome.

Elderly adults intrinsically exhibit reduced gut microbial diversity and impaired microbiota stability. Moreover, most older patients have comorbidities such as hypertension and diabetes and require long-term polypharmacy, which increases susceptibility to gut microbiota perturbations. These factors may further reduce tolerance to adverse drug reactions.

The delta over baseline (DOB) is the quantitative readout of the ^13^C-urea breath test (^13^C-UBT) and reflects intragastric *H. pylori* urease activity, which is commonly used as a surrogate for bacterial burden. In principle, higher DOB values may indicate greater *H. pylori* colonisation density [19]. Previous studies have reported lower eradication rates with increasing bacterial load [20], whereas several large-scale analyses in recent years suggest that the association between UBT values and eradication outcomes is not simply linear [21].

In this study, a moderate baseline UBT bacterial load emerged as an independent protective factor for successful *H. pylori* eradication, supporting a non-linear relationship between bacterial burden and treatment outcome. UBT values represent the integrated effects of urease activity and the gastric microenvironment. In older adults in particular, gastric mucosal atrophy, intestinal metaplasia, altered gastric acid secretion, and concomitant medications may all weaken the concordance between UBT readings and the true, eradicable bacterial load. Accordingly, a low UBT value does not necessarily indicate “mild infection”; it may also reflect reduced test signal or heterogeneity in infection distribution and/or gastric milieu, such that eradication is not intrinsically easier. Conversely, although the high-load group did not show a statistically significant difference, the corresponding confidence interval was relatively wide, suggesting limited statistical power due to sample size and/or the number of informative cases after stratification.

This study included antibody test results from 32 elderly patients, with a predominance of *H. pylori* Type I infection (78.13%), consistent with data from other clinical studies [22,23]. Further exploration of the association between *H. pylori*-related antibodies (CagA, VacA, UreA, and UreB) and composite antibody status with eradication rates showed no significant differences between positive and negative groups for individual antibodies. This result is likely closely related to the small sample size (only 32 cases), which reduced statistical power, making it difficult to accurately reflect true group differences and limiting the generalisability of the conclusions.

Overall trends showed slightly higher eradication rates in CagA- and VacA-positive patients compared to negative patients, aligning to some extent with conclusions from prior studies [24]. In the composite antibody analysis, the eradication rate in the VacA^+^/CagA^+^ double-positive group (100.0%) was markedly higher than that in the UreA^+^/UreB^+^ double-positive group (66.7%). However, given the small numbers (*n* = 3 and *n* = 6, respectively), this observation should be interpreted cautiously and requires confirmation in larger cohorts.

*H. pylori* exhibits substantial strain-level heterogeneity in pathogenic potential. The principal determinants of these differences are the repertoire, expression levels, and combinations of virulence genes. The key clinical value of virulence genotyping lies in refined stratification of bacterial virulence and disease risk, thereby informing individualised management. For patients infected with highly virulent strains, such as those carrying cagA (particularly East Asian strains with the EPIYA-D motif or Western strains with multiple EPIYA-C motifs), the vacA s1m1 genotype, or high-risk combinations (e.g., cagA^+^/vacA s1m1^+^) that are recognised markers of peptic ulcer disease, gastric mucosal atrophy, and gastric cancer [25,26], more intensive eradication strategies and strengthened long-term surveillance may be warranted. Conversely, in patients infected with low-virulence strains, individualised intervention and follow-up can be tailored by integrating clinical symptoms, histopathological changes in the gastric mucosa, family history of gastric cancer, and other relevant risk factors.

Eradication efficacy is closely related to antimicrobial resistance in the infecting strain to commonly used agents. Furazolidone is a key component of several eradication regimens, and resistance may substantially compromise treatment success. In addition, *H. pylori* resistance patterns show marked geographic heterogeneity. Although furazolidone is widely used in China and remains associated with low resistance rates in many regions [27], robust and up-to-date epidemiological data on the prevalence and temporal trends of furazolidone resistance among *H. pylori* isolates from the Beijing area are lacking. Accordingly, the potential impact of furazolidone resistance on eradication efficacy could not be quantitatively assessed in the present study.

The principal limitation of this study is the relatively small sample size, which reduced statistical power and precluded excluding residual confounding due to chance. This is particularly relevant to the virulence-factor analyses, in which we did not further stratify by other determinants of eradication outcomes, such as regimen type, the severity of gastric mucosal injury, or specific comorbidities. Furthermore, our cohort comprised elder adults only and did not include younger comparators; therefore, we could not perform cross-age comparative analyses or mechanistic exploration of age-related differences in treatment response. Finally, the lack of long-term follow-up limited the evaluation of eradication durability and subsequent clinical outcomes, including disease progression. Future studies with larger samples, broader enrolment, and extended follow-up are needed to define the long-term clinical value of different regimens.

## 4. Materials and Methods

This retrospective, observational, multicentre real-world study was conducted in accordance with the ethical principles outlined in the *Declaration of Helsinki* and was approved by the Medical Ethics Committee of Peking University First Hospital (Approval No. 2023Y009-001). All patients voluntarily attended the hospital for treatment and follow-up, with no intervention by researchers during this process. Researchers solely evaluated the participants’ treatment regimens and determined outcomes using the urea breath test.

Patients aged 60 years or older, diagnosed with *H. pylori* infection (confirmed by a positive ^13^C-urea breath test, 75 mg ^13^C-urea; Shenzhen Zhonghe Headway Bio-Sci & Tech Co., Ltd., Shenzhen, China) in the Peking University First Hospital, Peking University International Hospital, Dongzhimen Hospital of the Beijing University of Chinese Medicine, Guang’anmen Hospital of the China Academy of Chinese Medical Sciences, and Beijing Jishuitan Hospital were invited for study participation. The inclusion criteria were as follows: (1) confirmed *H. pylori* infection, indicated by a positive ^13^C-urea breath test; (2) age ≥ 60 years; (3) receiving and completing the full course of any amoxicillin-containing regimen (VA, RA, RJAF, or RBAF) and having no history of prior eradication therapy; (4) providing consent for the collection of clinical data and participation in follow-up.

The four treatment regimens were as follows: VA group: vonoprazan tablet, 20 mg, twice daily (bid), taken orally 30 min before morning and evening meals, and amoxicillin capsule, 1000 mg, three times daily (tid), taken orally after meals. RA group: rabeprazole, 20 mg, three times daily (tid), taken orally 30 min before meals, and amoxicillin capsule, 1000 mg, three times daily (tid), taken orally after meals. RJAF group: rabeprazole, 20 mg, twice daily (bid), taken orally 30 min before meals; Jinghua Weikang granules, 240 mg, twice daily (bid), taken orally 30 min before meals; amoxicillin capsule, 1000 mg, twice daily (bid), taken orally after meals; and furazolidone, 100 mg, twice daily (bid), taken orally after meals. RBAF group: rabeprazole, 20 mg, twice daily (bid), taken orally 30 min before meals; amoxicillin capsule, 1000 mg, twice daily (bid), taken orally after meals; furazolidone, 100 mg, twice daily (bid), taken orally after meals; and bismuth potassium citrate, 20 mg, twice daily (bid), taken orally 30 min before meals. All regimens were administered for a 14-day course.

Eradication of *H. pylori* was assessed using the urea breath test (UBT) at least 4 weeks after completion of antibiotic-based anti-*H. pylori* therapy and discontinuation of proton pump inhibitors (PPIs). Serum samples were collected from all patients before initiation of treatment for the detection of serum antibodies against *H. pylori* virulence genes.

Data were analysed using SPSS 26.0 software, and graphs were generated with GraphPad Prism 10. Measurement data conforming to a normal distribution were expressed as mean ± standard deviation ($\bar{x}$ ± s), and inter-group comparisons were performed using one-way analysis of variance (ANOVA). Data with a non-normal distribution are expressed as median (interquartile range) [M (Q1, Q3)], and inter-group comparisons were conducted using the Kruskal–Wallis H test. Count data are expressed as number (percentage) [*n* (%)], and inter-group comparisons were made using the Chi-square (χ^2^) test or Fisher’s exact probability test. Ordinal data were analysed using the Kruskal–Wallis H test. A *p*-value < 0.05 was considered statistically significant. Multivariate logistic regression analysis was performed to identify independent factors associated with *Helicobacter pylori* eradication, and the model’s goodness of fit was evaluated using the Hosmer–Lemeshow test. A *p*-value < 0.05 was considered statistically significant.

## 5. Conclusions

In elderly adults with *H. pylori* infection, dual therapy demonstrates non-inferior efficacy compared with triple therapy and conventional quadruple therapy. More complex regimens do not appear to provide incremental clinical benefit. The specific effects of baseline ^13^C-urea breath test (UBT) values and *H. pylori* virulence factors on eradication outcomes require further confirmation in large, multicentre studies with more in-depth analyses.

## Figures and Tables

**Table 1:** Comparison of baseline data in the four groups.

	VA (*N* = 80)	RA (*N* = 84)	RJAF (*N* = 67)	RBAF (*N* = 68)	X^2^/F/H	*p*-Value
**Age (*x* ± *s*, years)**	65.99 ± 5.09	65.56 ± 4.29	64.57 ± 4.55	65.38 ± 3.83	F = 1.265	0.287
**Gender (*n*, %)**					X^2^ = 5.305	0.151
**Men**	35 (43.75%)	40 (47.62%)	21 (31.34%)	33 (48.53%)		
**Women**	45 (56.25%)	44 (52.38%)	46 (68.66%)	35 (51.47%)		
**Comorbidity (*n*, %)**					X^2^ = 32.113	0.057
**No underlying diseases**	13 (16.3%)	19 (22.6%)	20 (29.9%)	16 (23.5%)		
**One underlying disease**	20 (25%)	11 (13.1%)	13 (19.4%)	11 (16.2%)		
**Multiple underlying diseases**	47 (58.75)	54 (64.28%)	34 (50.74%)	41 (60.29%)		
**Baseline UBT value [*M* (*Q1*, *Q3*), ‰]**	19.5 (11.15, 25.15)	20.15 (13.75, 38.98)	23.2 (14.3, 42.8)	21.84 (9.7, 38.9)	H = 5.334	0.149

Footnote: VA regimen: amoxicillin (1 g, tid) and vonoprazan (20 mg, bid); RA regimen: amoxicillin (1 g, tid) and rabeprazole (20 mg, tid); RJAF regimen: amoxicillin (1 g, bid), rabeprazole (20 mg, bid), Jinghua Weikang granules (240 mg, bid), and furazolidone (100 mg, bid); RBAF regimen: amoxicillin (1 g, bid), rabeprazole (20 mg, bid), bismuth potassium citrate (20 mg, bid), and furazolidone (100 mg, bid). All regimens were administered for a 14-day course.

## Data Availability

The datasets used during the current study are available from the corresponding author upon reasonable request, with approval from the Ethics Committee of Peking University First Hospital.
